# The hypodermic syringe performance based on the ISO 7886-1:2017: A narrative review

**DOI:** 10.1097/MD.0000000000031812

**Published:** 2022-12-09

**Authors:** Krisdiyanto, Raja Ariffin Bin Raja Ghazilla, M. Azuddin, Muhammad Khairi Faiz Bin Ahmad Hairuddin, Muhammad Akhsin Muflikhun, Nurvita Risdiana, Eki Afifuddin

**Affiliations:** a CPDM, Department of Mechanical Engineering, Faculty of Engineering, University of Malaya, Kuala Lumpur, Malaysia; b Mechanical Engineering Department, Faculty of Engineering, Universitas Muhammadiyah Yogyakarta, Yogyakarta, Indonesia; c Mechanical and Industrial Engineering Department, Faculty of Engineering, Universitas Gadjah Mada, Yogyakarta, Indonesia; d Department of Mental Health Nursing, School of Nursing, Universitas Muhammadiyah Yogyakarta, Yogyakarta, Indonesia.

**Keywords:** dead space, fit plunger, force operation, ISO 7886-1, leakage, syringe

## Abstract

A syringe is used to inject fluid or medicine into the patient’s soft tissue. The main components of the syringe were the needle, barrel, and plunger. The use of syringes in the medical world is relatively high, and especially since the COVID-19 pandemic, the use of hypodermic syringes increased sharply due to vaccination. The syringe used must be effective and of good quality, so the International Organization for Standardization (ISO) has published test procedures and minimum specifications for hypodermic syringes. The performance of the syringe can be observed from the dead space, force piston operation, water and air leakage, and fitting position of the plunger in the barrel. This review shows that most researchers use the weighing method to measure the dead space, although some use other methods. The researchers found that most of the products met the minimum specifications of the ISO, and that the dimensions and shape of the syringe affected the dead space. Researchers have not examined other performance measures recommended by the ISO. Researchers have focused more on force injection than force piston operation, leakage after injection or back spray than air and water leakage, and reduction the friction of the plunger without considering the fitting position of the plunger in the barrel.

## 1. Introduction

Syringes are among the most important tools in the medical field. Disposable syringes are one of the most frequently used medical devices for intravenous or intramuscular injections.^[1]^ A syringe is used to inject the drug into the patient’s body.^[2–11]^ As early as 1656 and 1657, Christopher Wren, a professor of astronomy, experimented with the process of injecting drugs directly into the veins of animals and human beings. Wren’s method uses a quill feather-pen with an attached bladder. The discovery of an 1834 engraving showing the poet Thomas Campbell holding a workable hypodermic syringe offers a mysterious report of its development, since no medical texts note the existence of such early use. While crude transfusing of blood and drug administration did exist, the forerunners of the modern hypodermic syringe would not arrive until developments by Irish surgeon F. Rynd in 1844 and Scottish physician Alexander Wood in 1853.^[12]^

Wood modified the needle to reach a fine tip, which facilitated its penetration into tissues. Syringes were made in 1870 by Dental Cosmos (USA), where it was mentioned that they were made of hardened steel but did not mention their caliber and size. In 1920, syringes were manufactured using stainless steel.^[13]^

**Figure 1.**
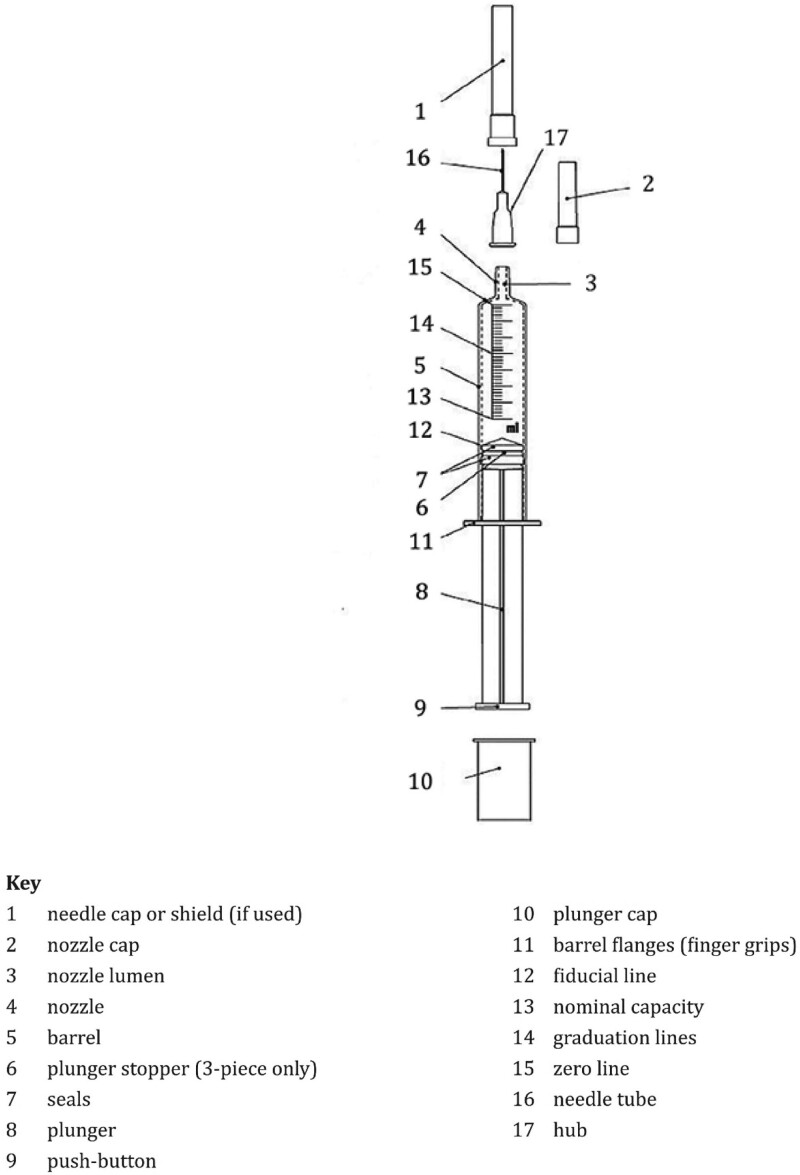
Schematic representation of hypodermic syringe for single-use.^[24]^

The syringe consists of three main components of the syringe that is the barrel, plunger, and needle.^[14–23]^ Of the 3 main components, the syringe is composed of several components as shown in Figure 1. There are many variations in the syringe material; most needles use stainless steel, titanium, and other coated materials,^[25]^ and the majority of plunger and barrel materials are polypropylene.^[26–30]^

In the past few years, the medical world has required many syringes; 16 billion syringes are consumed every year,^[31]^ and the sales estimation between 2013 and 2020 increased by about $3 billion.^[32]^ The other researcher shows the prediction of an annual growth rate of 13.8 % between 2012 and 2018.^[33]^ Pandemic COVID-19 has increased the use of hypodermic syringes for vaccine.^[34]^

The increase in the number of syringes that occur in the medical world will certainly greatly affect the need for and manufacture of syringe, which must be maintained because hypodermic syringes are the first point of contact with biological systems during injection and therefore must be made from biocompatible materials that are pharmacologically inert, sterilizable and nontoxic. The hypodermic syringe must be free from defects that disturb performance, and safety standards for the use of syringes have been regulated in IS0 7886-1: 2017. International Organization for Standardization (ISO) 7886-1 is the second edition of the international standard for the use of syringes, and several changes have been made including the test method for syringes and the test method to determine the force required to operate the piston. Based on the security standards that have been regulated in ISO, several performance tests have been regulated, the syringe performance test consists of dead space, freedom from leakage, operating piston force, and fit of the plunger in the barrel.^[24]^ Syringe type affects the dead space,^[2,6,35–42]^, operating force^[23,39,43–46]^, and leakage^[47–49]^. In addition to dead space testing, freedom from air and liquid leaked past the plunger stopper, forced the piston to operate, and fit the plunger stopper/plunger in the barrel, which are of concern in the use of syringes such as those carried out by Struik et al They compared the performance of a single-use syringe versus a multi-use MR contrast injector by reviewing the preparation time, cost of disposables, volume of contrast material used for single-use and a multi-use (MI) MR contrast injector^[50]^. The ISO determines the performance testing and minimum specification of performance in document ISO 7886-1 which has the title Sterile hypodermic syringes for single use^[24]^.

## 2. Method of paper screening

The researchers searched scientific journals from Google Scholar using the following keywords: syringe performance, syringe dead space, force, syringe leakage, and syringe fit plunger. The addition of sources in the review paper adds the scientific journal used as a reference for the search results. Additional sources were journals near the topic object of the research.

## 3. Results

### 3.1. Dead space

The maximum dead space and the procedure for testing the dead space of a hypodermic syringe were determined by the ISO 7886-1. The recommendations for the maximum dead space on a hypodermic syringe are presented in Table 1. The procedure for testing the dead space on a hypodermic syringe involves weighing an empty and dry syringe, and then filling the hypodermic syringe with distilled water. Water was expelled by fully pressing the plunger and wiping the outer syringe, and the syringe was reweighted. The different mass of the syringe is the residual fluid, that is the conversion of mass to the volume of distilled water.

**Table 1:** Maximum dead space based on ISO 7886-1.^[24]^

Syringe volume (mL)	Maximum dead space (mL)
*V* < 2	0.07
2 ≤ *V* < 5	0.07
5 ≤ *V* < 10	0.075
0 ≤ *V* < 20	0.10
20 ≤ *V* < 30	0.15
30 ≤ *V* < 50	0.17
*V* ≥ 50	0.20

ISO = International Organization for Standardization, V = volume.

The ISO recommends that the maximum dead space is 0.07 mL for a barrel volume below 5 mL, 0.075 mL for a 5 mL barrel volume < *V* < 10 mL, 0.10 mL for a 10 mL barrel volume < *V* < 20 mL, 0.15 mL for a 20 mL barrel volume < *V* < 30 mL, 0.17 mL for a 30 mL barrel volume < *V* < 50 mL, and 0.20 mL for a volume barrel of more than 50 mL.^[24]^ The available studies found potential variability in low-dose delivery volumes owing to dead space and technical limitations associated with the measurement of a small volume of liquids.^[51]^ Dare et al conducted this test to determine the Covid 19 vaccine needle and syringe dead space volumes, because the difference in the dose that the syringe was able to withdraw, so it was suspected that this was due to the difference in dead space volume in different needles and syringes.^[50]^ The dead space volume test was carried out according to the standard determined by the in ISO 7886-1, the results showed that the highest average dead space was obtained in a 3 mL syringe with an average dead space of 0.075 with a standard deviation of 0.0105, which exceeded the standard set by the in ISO 7886-1 (2017). A high volume dead space value will certainly affect the effectiveness of the use of syringes, utilizing needles and syringes with low dead space clearly helps to increase efficiency by reliably obtaining the 6th dose of vaccine per multidose vial.^[50]^

Result of measuring dead space volume which shows that the maximum dead space that occurs is 0.0750 mL, and the lowest dead space volume is 0.0104. Knowing the dead space volume will help the use of syringes more effectively and increase vaccine supply, the data is shown in Table 2. Accurate dosing of the right medicine to the right patient is a key element of safe and efficacious pharmacotherapy, yet prone to technical challenges and human error when dosing involves the administration of a small volume of liquid medicineene.^[52]^ Dare et al conducted a study to increase the supply of vaccines to prevent the transmission of covids with low dead volume syringes and needles to determine which medical devices allow extraction of doses from different vaccine vials (Pfizer-BioNTech, AstraZeneca, Moderna, and Johnson & Johnson Vaccine). According to his study, syringe accuracy is important and can compromise the chance of extracting additional doses when withdrawing too large a volume.^[6]^ According to study by Dare et al, the dead space of the syringe or needle combination is the determining factor for extraction from the vaccine vial. Although the ISO has provided procedures to test the dead space of hypodermic syringes, some researchers have used another method. The kappa coefficient was used to measure agreement between the interview results of the injection process in 6 groups in Kulob and 6 in Khorog, Tajikistan, with a total of 100 participants. Participants injected heroin into their bodies. The most popular syringes in this group were 2 and 5 mL, and the needle size was 25 to 21, with a length of 25 mm and diameter of 25 gauge (G). The kappa coefficient and the percentage agreement showed that the overall evaluation of the low dead space syringe was 0.91 and 99.95 %, the inter-rater reliability was excellent, and the lowest dead space was a 1 mL insulin syringe with 12 mm 28 G permanently attached needle.^[36]^ Other studies have investigated dead space syringes (auto-disable syringes and syringes used with intradermal adapters), Luer-slip needles and syringes, miniature syringes, hollow microneedle devices, and associated disposable jet injectors. These devices were used to withdraw 0.1 mL of a fractional dose from a single-dose IM glass vial, which is then dispensed. The vials and syringes are weighed. The mean dead space of the tested devices ranged from 3.2 μL to 96.7 μL per injection, and the syringe with the needle inserted had a low dead space.^[41]^ Other studies have used the gravimetric method or analytical method to measure the mass of ions in new and dry syringes. The specimens used in this study were 56 combinations of needles and syringes obtained from needles and syringes, with barrel capacities ranging from 0.5 to 20 mL and needle lengths ranging from 8 to 38 mm. The mean dead space was 3 μL on a low dead space syringe with a permanently attached needle, 13 L on a high dead space syringe with a low dead space needle, 45 μL on a low dead space syringe with a high dead space needle, and 99 μL.^[53]^ Overall, the above research shows that the syringe dead space is excellent and meets the requirements of the Organization for Standardization, however, an experiment shows that some brands are not qualified. The experimental method was based on the Organization for Standardization. For example, dead spaces 1 and 2.5 mL were less than 0.07 mL, 4 of the 5 brands of 5 mL syringes were greater than 0.075, and one of 5 brands of 10 mL was greater than 0.1 mL.^[2]^

**Table 2:** Dead space volume of vaccination supplies.^[50]^

Vaccination product	Specific details	Mean dead space (mL)	Standard deviation (mL)
Fixed needle and syringe combinations	VanishPoint® 25G × 1with 1 mL syringe	0.0103	0.0049
	VanishPoint® 23G × 1with 3 mL syringe	0.0750	0.0105
	BD® 1 mL syringe without needle	0.0343	0.0114
Syringes	BD® 3 mL syringe without needle	0.0179	0.0110
	DPS® 1 mL syringe without needle	0.0104	0.0029
	Magellan® 23G × 1needle without syringe	0.0309	0.0097
	Magellan® 25G × 1needle without syringe	0.0478	0.0015
Needles	SolCare® 25G × 1needle without syringe	0.0652	0.0042
	DPS® 23G × 1.5needle without syring	0.0668	0.0070
	TKMD® 21G × 1.5needle without syring	0.0742	0.0013

G = gauge.

### 3.2. Force operation

**Figure 2.**
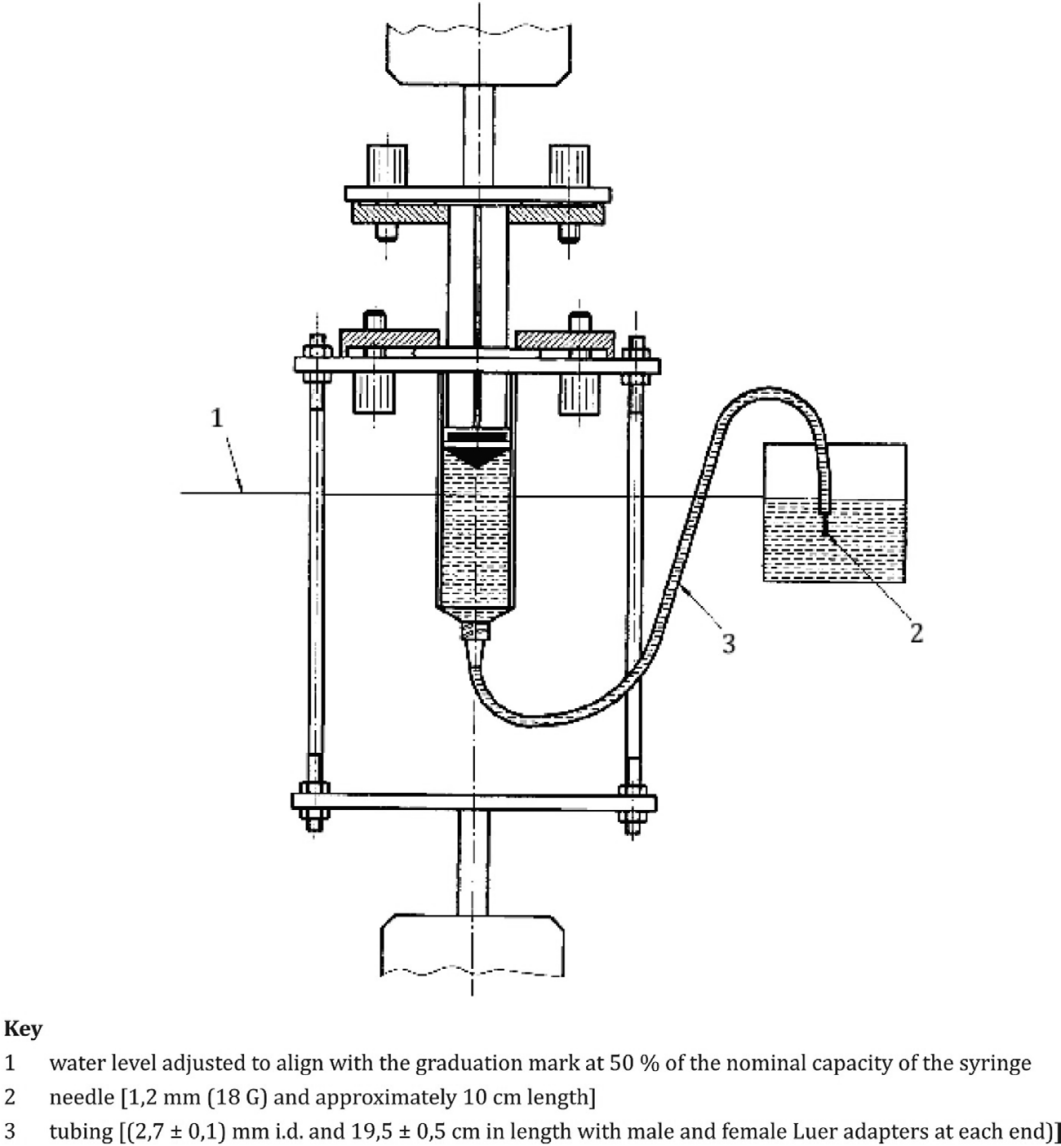
Operating force testing scheme.^[24]^

The second indicator of syringe performance is the force operation. The procedure for testing the force operations is described in the ISO standards. The operating force testing scheme based on ISO 7886-1 is presented in Figure 2.

In the operating force test, the water level in the reservoir was set at half the nominal capacity of the syringe, and the needle dimension were 18 gauge and approximately 10 cm. The internal diameter is 2.7 + 0.1 mm and the length 19.5 + 0.5 cm with male and female Luer adapters at each end.

The syringe is filled with water and held vertically; the piston body must not move because of its weight.^[20]^ The force for the movement of the piston body and filling water in the syringe or discharging the water from the syringe was determined by a mechanical experiment machine. A mechanical experimental machine was also used to determine the force required to flow the water. Operating force testing was also carried out by Busillo and Colton to test fluid flow as an indicator in the characterization process of plastic hypodermic needles to determine the amount of force that can be recommended for the operation of plastic hypodermic needles.^[54]^ Studies have shown that the penetration force on the skin and polyurethane rubber is linearly related, and that needle insertion without silicone lubrication increases the penetration force and bleeding after withdrawal of the needle.^[55]^ The strong force effect along with the steadiness of the personnel injecting the solution physically complicates pain stimulation.^[56]^ The researchers tested the operating force required to obtain a product marketed to a specific group. For example, Sheikhzadeh et al tested the design of a syringe for patients with rheumatoid arthritis. Researchers measure the force using a force-measuring device placed on the patient. The study also compared 2 syringes with different design of the plunger and barrel will need different injection force required^[44]^ Other researchers have also made innovations that can help patients reduce force injection, so the researcher produced a syringe adapter that can reduce muscle strain and fatigue even though the use of the device will increase the operating time by 1.5 times.^[43]^ In addition, the difference in the shape of the needle tip affects the force used during penetration,^[39,45]^. For example, the 5-bevel tip needle requires less penetrating force than the 3-bevel tip needle^[45]^, and the newest needle also results in less penetration^[39]^. Prasetyono et al conducted a laboratory study on the measurement of injection force with different combinations of syringes and needles to determine the initial strength and maintenance force of the combination of needle and syringe as a reference for the anesthetic process. The experimental process was carried out by combining syringes measuring 1 mL, 3 mL, 5 mL, 10 mL, and 20 mL with the original needle, 27 G, 27 G spinal, and 30 G in which each combination was tested for compression three times to measure the initial and maintenance forces, it determined which needle and syringe combination had the lowest initial force so that it could be a reference for pain-free injections. Overall, the design affects the syringe force operation and additional equipment can be used to reduce the force operation. In addition, the shape of the needle tip affected the penetration force.

### 3.3. Leakage

The 3rd parameter of performance, based on the standards of the Organization for Standardization is leakage. There are 2 types of leakage: air and liquid leakage.^[24]^ Karpuz and Ozer stated that plastic syringes are class 1A medical devices that are used frequently for the parenteral administration of drugs, and leakage is one of the factors that must be tested and complied with according to the requirements that have been determined, the test procedure in the standard from the ISO shows that the testing is used to identify the leakage at the sealing plunger.

**Figure 3.**
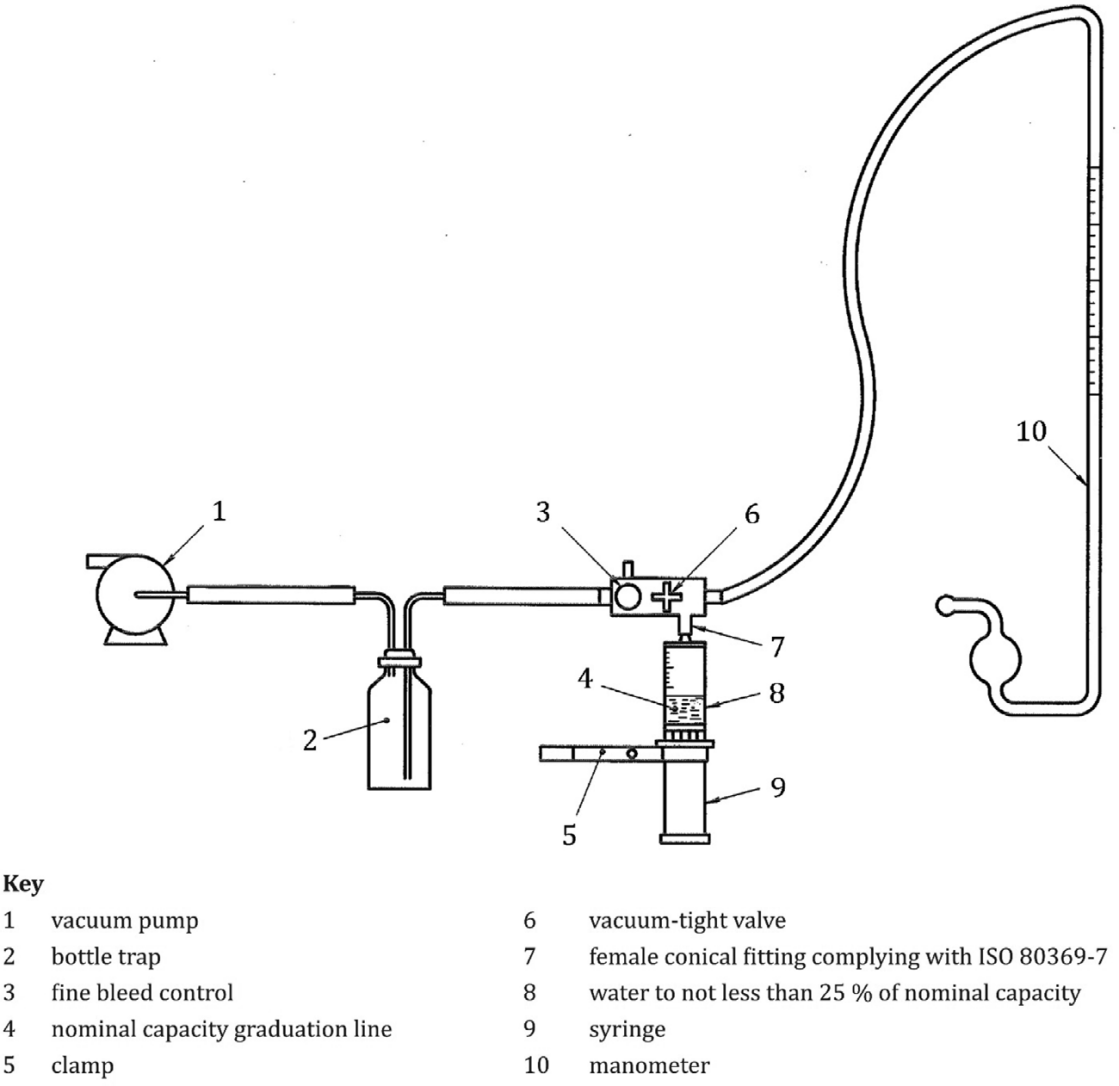
Air leakage testing scheme on the hypodermic syringe.^[24]^

Water leakage testing on the hypodermic syringe is done by filling the syringe in nominal capacity with distilled water and giving a sideways force of 0.25 to 3 N at the end of the plunger, and then the plunger is pressed so that the internal pressure on the syringe is 200 to 300 kPa. Pressure was maintained for 30 to 35 seconds to ensure no leakage. The procedure for air leakage testing is different from that for water leakage testing. The test scheme of air leakage for the hypodermic syringe is shown in Figure 3.

The steps for testing air leakage are that the contents of the hypodermic syringe are not more than 25% of capacity, then pull the plunger to the fiducial line from the nominal capacity. The syringe was attached to the test equipment as in the air leakage test scheme, and a vacuum is generated until the manometer read 88 kPa below the ambient atmospheric pressure. Observation required 60 seconds to ensure that the air leakage and pressure changes in the manometer were recorded.

An experiment was conducted to investigate the effect of the Teflon-coated rubber in a glass barrel by mathematical analysis. The result of this experiment is that besides silicon oil, the leak rate depends on the interfacial surface roughness, and Teflon surface deformation can reduce the leak rate.^[57]^ On the other hand, there are three experiments on liquid leakage, but the researcher focuses on leakage after injection. Chan et al investigated the effect of the air bubble pressure at the headspace when the needle was removed. The investigation showed that the pressure measurement indicated a decreasing pressure when the needle was removed after injection.^[48]^ In another study, Varden et al investigated post-injection leakage in the intervertebral disc, the goal of which was to establish a protocol for quantifying post-injection leakage and to test its sensitivity to factors believed to affect needle track geometry. The needle diameter is significantly affected by the maximum volume prior to leakage, and one of the factors that cause leakage after needle retraction is axial compression.^[56]^ Other researchers have also investigated liquid leakage after injection. Præstmark et al studied the injection technique and needle design to minimize fluid leakage. This investigation used a clinical study to determine leakage. The variables used in this study were injection volume, speed, injection region, needle wall thickness, needle taper, injection angle, and wait time from the end of the injection to the withdrawal of the needle from the skin. The results showed the volume of injection to avoid leakage was 800 *μ*L at a time; the injections in the abdomen caused less leakage than the thigh injections; a 32 G needle caused less leakage than a 31 G needle and a 32 G tip needle; needle insertion angle of caused less leakage than an angled 45° and below the insertion, and the minimum time of needle withdrawal was 3 seconds.^[47]^ Kenneth et al also investigated the effect of needle dimensions on liquid leakage. In addition to the needle dimensions, the researcher group also investigated the effect of barrel volume. The researchers used 25 G, 22 G, and 20 G needles, or the dimensions were different from those used in the experiment by Præstmark et al The fluid was injected into fresh porcine intestines, which had dimensions of 5 and 10 cm. Injections with 22-gauge needles reduced the frequency of leakage, whereas 20 mL instilled in 5 cm segments increased the frequency of leakage in intact segments of the porcine jejunum.^[49]^ The researchers above showed that air and water leakage research is not based on standards from the ISO.

### 3.4. Fit of the plunger in the barrel

The fourth indicator of hypodermic syringe performance was the fit position of the plunger in the barrel. The installation of the plunger in the barrel affects the leakage that occurs in the syringe. The position of the plunger should have no movement because of the force from the mass of water in the barrel when the syringe is in a reserved position with a nominal capacity of water.^[24]^ The syringe was filled with distilled water at nominal capacity and held vertically with the first end, and then the syringe was rotated and the first 1 was positioned in the uppermost position. The mass of water above plunger and the plunger was not moved by the force from the fluid mass.

This fourth syringe performance indicator is rarely studied or discussed in scientific journals. Researchers have focused on reducing the friction between the seal plunger and barrel without testing the friction to maintain the plunger position. Low friction combined with smooth movement is a vital characteristic of drug delivery devices, particularly medical syringes. Surface roughness has a significant influence on manyphysical phenomena such as adhesion, friction, contact mechanics, and seal leakage.^[48]^ Friction is also affected by the texture of the plunger^[58]^ and lubricant.^[59]^ The review showed that the researchers did not investigate the fit plunger position of the barrel. Performance testing at the plunger position can also be said to have been represented by leak testing of the syringe and the operating style of the syringe.

## 4. Conclusion

The results of this review show that the ISO published a document explaining the performance testing procedure of a hypodermic syringe. In addition to the performance-testing procedure, this document also provides the minimum specifications of the performance-testing values for hypodermic syringes. Performance testing of the hypodermic syringe consists of testing the dead space, force piston operation, leakage, and fitting the plunger in the barrel. In general, researchers have investigated the effect of the dimensions and shape of syringes on the dead space. Most products met the ISO criteria, although some products were not met. Most researchers use weighting as the ISO procedure. By knowing the value of dead space, syringe users can determine the optimal syringe so that they can maximize the use of the dose to be used. However, some researchers have used the kappa coefficient to determine the dead space value of the syringes. While the performance of force piston operation suggested by the ISO is still little studied by researchers, some researchers use this test standard to determine the force used in a new syringe design or determine the appropriate force for optimal needle operation. Researchers generally focus more on the injection force; therefore, many studies have shown that it is related to the force used when injecting the needle into the soft tissue, one which is to reduce pain in the injected area. In addition, researchers have not focused on water and air leakage to force piston operation, and most researchers focused on the material used on the plunger surface to reduce friction and prevent barrel leakage due to friction between the plunger and barrel. On the other hand, the researchers also did not focus on water and air leakage, which the International Organization mentions for standardization. Researchers have focused on leakage after injection or back spray. The last performance based on the ISO is the fitting position of the plunger in the barrel. This performance has also attracted the attention of researchers because many have focused on reducing the friction between the seal plunger and barrel without testing the friction to maintain the plunger position.

## Author contributions

**Conceptualization:** Krisdiyanto, Nurvita Risdiana.

**Data curation:** Nurvita Risdiana, Eki Afifuddin.

**Investigation:** Nurvita Risdiana.

**Resources:** Nurvita Risdiana.

**Supervision:** Raja Ariffin Bin Raja Ghazilla, M. Azuddin, Muhammad Khairi Faiz Bin Ahmad Hairuddin, Muhammad Akhsin Muflikhun.

**Validation:** Nurvita Risdiana.

**Writing – original draft:** Krisdiyanto, Eki Afifuddin.

**Writing – review & editing:** Krisdiyanto, Eki Afifuddin.
